# Mental health in individuals with severe mental disorders during the covid-19 pandemic: a longitudinal investigation

**DOI:** 10.1038/s41537-022-00225-z

**Published:** 2022-03-08

**Authors:** Alex Hofer, Timo Kachel, Barbara Plattner, Anna Chernova, Andreas Conca, Martin Fronthaler, Christian Haring, Bernhard Holzner, Markus Huber, Josef Marksteiner, Carl Miller, Silvia Pardeller, Verena Perwanger, Roger Pycha, Martin Schmidt, Barbara Sperner-Unterweger, Franziska Tutzer, Beatrice Frajo-Apor

**Affiliations:** 1 Division of Psychiatry I, Department of Psychiatry, Psychotherapy, Psychosomatics and Medical Psychology, Medical University Innsbruck, Innsbruck, Austria; 2 Department of Psychiatry, Sanitary Agency of South Tyrol, General Hospital of Bolzano, Bolzano, Italy; 3 Sanitary Agency of South Tyrol, Therapy center Bad Bachgart, Rodengo, Italy; 4 Department of Psychiatry and Psychotherapy B, State Hospital Hall in Tyrol, Hall in Tyrol, Austria; 5 Department of Psychiatry, Sanitary Agency of South Tyrol, General Hospital of Brunico, Brunico, Italy; 6 Department of Psychiatry and Psychotherapy A, State Hospital Hall in Tyrol, Hall in Tyrol, Austria; 7 Department of Psychiatry, County Hospital Kufstein, Kufstein, Austria; 8 Department of Psychiatry, Sanitary Agency of South Tyrol, General Hospital of Merano, Merano, Italy; 9 Department of Psychiatry, Sanitary Agency of South Tyrol, General Hospital of Bressanone, Bressanone, Italy; 10 Department of Psychiatry, County Hospital Lienz, Lienz, Austria; 11 Division of Psychiatry II, Department of Psychiatry, Psychotherapy, Psychosomatics and Medical Psychology, Medical University Innsbruck, Innsbruck, Austria

**Keywords:** Psychosis, Schizophrenia

## Abstract

Research on the long-term mental health impact of the COVID-19 pandemic across mental disorders is limited, and information on the impact of public health policy measures with varying strictness is missing. This study therefore aimed at investigating psychological distress among residents of Tyrol (Austria) and South Tyrol (Italy) at the early stages of the pandemic and 5 months thereafter and examined how sociodemographic, protective, and risk factors relate to change over time. One hundred and fifteen people with severe mental illness (SMI; schizophrenia spectrum disorder, bipolar disorder, major depressive disorder with psychotic features) or major depressive disorder without psychotic features (MDD) and 481 community controls without mental disorders participated in an online survey. Next to the collection of sociodemographic and COVID-19 related variables, the Brief Symptom Checklist, the Resilience Scale, the Multidimensional Scale of Perceived Social Support, the Three-Item Loneliness Scale, and the Multidimensional State Boredom Scale-Short Form were used to assess psychological distress, resilience, perceived social support, loneliness, and boredom. Levels of psychological symptoms and the prevalence of psychological distress were significantly higher in individuals with MDD compared to the other two groups, and Italian participants were more prone to anxiety than those from Austria. Psychological distress was predicted by a lower degree of both resilience and perceived social support as well as loneliness and boredom. Notably, the prevalence of clinically relevant psychological symptoms remained unchanged among each group over time. These results underscore the relevance of tailored prevention and mitigation strategies to meet the specific needs of people both with and without mental disorders.

## Introduction

The COVID-19 pandemic and related public health policy measures including quarantine, lockdown, and physical distancing are largely affecting mental health worldwide. Previous studies have consistently detected a high prevalence of anxiety, depressive symptomatology, and psychological distress among the general population^1^, however, the pandemic has been suggested to have an even more detrimental effect on individuals with a pre-existing psychiatric disorder^2^ and in particular, those with severe mental illnesses (SMI) such as schizophrenia or bipolar disorder (BD). They may not only be more susceptible to infection and to suffer complications of COVID-19 but may also experience symptom exacerbations or worsening of the illness course due to increased stress, reduced social interactions, and disruptions in treatment delivery and availability^3^. However, studies in these populations and especially longitudinal data on the impact of the pandemic on mental health among individuals with pre-existing SMI are surprisingly rare and controversial. For example, a survey conducted in India found that 30% of 132 individuals with SMI reported a relapse of symptoms during COVID-19 lockdown^4^, while the severity of affective experiences and psychotic symptoms remained stable among a comparable sample from the United States^5^.

During the first months of the pandemic, a number of studies found higher levels of depression, anxiety, and stress in patients suffering from SMI compared to healthy control subjects^6–11^. Yocum et al.^12^, in turn, detected a lesser increase in distress in individuals with BD than in healthy subjects, although patients were more impacted by the COVID-19-related stay-at-home orders and recovered more slowly. It has to be noted, however, that pre-COVID-19 baseline measures are often lacking and that causal relationships between the pandemic and people’s mental health can frequently not be deduced from the available data. Of note, a recently published study that had used longitudinal data from three Dutch psychiatry case-control cohorts revealed higher levels of symptoms of depression, anxiety, worry, and loneliness among people with depressive, anxiety, or obsessive-compulsive disorders compared to individuals without mental health disorders, both before the pandemic and 2–8 weeks after the national lockdown in the Netherlands. In addition, symptom severity had increased more in people with no or less severe or chronic mental health disorders^13^. On the other hand, studies conducted among older adults suffering from major depressive disorder (MDD) or BD found fewer psychiatric symptoms during the first months of the COVID-19 outbreak when compared to baseline^14,15^, and it remains to be seen, whether and how these findings may change in the long-term.

Research on a potentially differential mental health impact of the COVID-19 pandemic across severe mental disorders is generally limited and equivocal. While a number of studies found that people suffering from schizophrenia spectrum disorders (SSD) experienced less COVID-19-related stress relative to those with affective disorders^9,10,16^, Pinkham et al.^9^ detected no group differences in this regard. Similarly, comparative studies of individuals with either MDD or BD also came to divergent results. Di Nicola et al.^17^, for example, found that a diagnosis of MDD specifically predicted the severity of psychological distress, whereas Van Rheenen et al.^11^ reported on higher levels of stress, depression, and financial concern among people with BD. Lastly, the psychological reaction to the COVID-19 lockdown in Spain was more pronounced among people suffering from depression or anxiety disorders compared to those with psychotic disorders or BD^7^. Despite a considerable overlap in terms of symptoms, familial patterns, risk genes, and outcome, it thus remains unclear whether the levels and changes in COVID-19-related psychological distress differ between individuals suffering from SMI as defined by Kessler et al.^18^ (SSD, BD, MDD with psychotic features) and those with MDD without psychotic features (referred as MDD in the rest of this manuscript). Conversely, a better understanding of the psychological impact of the COVID-19 pandemic on people with various pre-existing mental health disorders and knowledge about predictive factors influencing the extent of psychological burden on different patient groups facilitates the development of appropriate preventive measures and effective treatment strategies.

The main objective of the present longitudinal study was to investigate the impact of the COVID-19 pandemic on mental health of people with and without a history of mental disorders living in Tyrol (Austria) and South Tyrol (Italy). Though an inter-state border separates the region since 1919, the population has similar characteristics and is comparable in many ways (socioeconomic context, healthcare system, etc.)^19^. However, due to rapid dissemination of SARS-CoV-2 and an overstrained national healthcare system, COVID-19-related public health policy measures have been substantially stricter in Italy than in Austria. While both countries have intermittently suspended social and sports events as well as religious ceremonies in the course of the pandemic and have closed schools, universities, theatres, etc., further restrictions persisted considerably longer in Italy than in Austria. These public health policy measures included parks, playgrounds, and public green being closed down, and movement across the country being restricted. In addition, in Italy, machine gun soldiers patrolled city streets and a decree provided sanctions of up to 3 months in prison for those who violated the lockdown. Indeed, up to the end of the baseline (follow-up) survey of the current study, 4967 (43,630) SARS-CoV-2 cases (active + recovered) and 108 (528) deaths related to COVID-19 were recorded in Tyrol (~760,000 inhabitants)^20^, whereas 21,200 (69,419) cases and 469 (1138) deaths were registered In South Tyrol (~533,000 inhabitants)^21^. Accordingly, a secondary objective of this study was to explore whether psychological outcomes and well-being differed between countries. Here we report psychological distress at the early stages of the pandemic and 5 months thereafter in (1) individuals suffering from SMI or (2) MDD and (3) community controls with no self-reported mental health disorders. We also examined how sociodemographic, protective (resilience, perceived social support), and risk factors (loneliness, boredom) relate to change over time in those three groups.

## Results

### Baseline characteristics and COVID-19-related aspects

Table 1 depicts baseline characteristics of individuals suffering from SMI or MDD and the control group (controls). Compared to controls, SMI and MDD patients reported less educational years, being more often single and retired, and having a lower household income. Furthermore, SMI patients had more severe physical health problems (also in comparison to MDD patients) and smaller flat sizes, whereas MDD patients were older (same tendency for SMI patients) and more often unemployed.

**Table 1:** Baseline characteristics.

	SMI (*N* = 46)	MDD (*N* = 69)	Controls (*N* = 481)
Variable	Mean ± SD or *N* (%)	Mean ± SD or *N* (%)	Mean ± SD or *N* (%)
Gender			
Male	19 (41.3%)	28 (40.6%)	139 (28.9%)
Female	27 (58.7%)	41 (59.4%)	342 (71.1%)
Age (Years)	48.9 ± 14.3 (22–81)	48.5 ± 13.9 (22–82)^d^	44.3 ± 13.6 (18–96)
Education (Years)	13.7 ± 3.4^e^	12.7 ± 3.7^e^	15.6 ± 3.7
Residence			
Tyrol (Austria)	27 (58.7%)	44 (63.8%)	282 (58.6%)
South Tyrol (Italy)	19 (41.3%)	25 (36.2%)	199 (41.4%)
Relationship			
Single	21 (45.7%)^a^	27 (39.1%)^a^	91 (18.9%)
Fixed partnership	25 (54.3%)^c^	42 (60.9%)^c^	390 (81.1%)
Work situation			
Full-time	5 (10.9%)^c^	13 (18.7%)^c^	231 (48.2%)
Part-time	9 (19.5%)	7 (10.1%)^c^	113 (23.4%)
Self-employed	2 (4.3%)	3 (4.3%)	34 (7.1%)
Education/training	1 (2.2%)	5 (7.2%)	27 (5.6%)
From home	0 (0.0%)	1 (1.4%)	4 (0.4%)
Short-time work	2 (4.3%)	1 (1.4%)	4 (0.8%)
Unemployed	2 (4.3%)	8 (11.6%)^a^	4 (0.4%)
due to COVID-19	0 (0.0%)	2 (2.9%)	1 (0.2%)
Retired	21 (45.7%)^a^	22 (31.8%)^a^	43 (9.0%)
Homemaker	2 (4.3%)	1 (1.4%)	5 (1.0%)
Others	1 (2.2%)	3 (4.3%)	7 (1.5%)
Household income			
<25,000 €/year	34 (73.9%)^a^	43 (62.3%)^a^	160 (33.3%)
25,000–49,999 €/year	8 (17.4%)^c^	11 (15.9%)^c^	176 (36.6%)
≥50,000 €/year	2 (4.4%)^c^	6 (8.7%)^c^	136 (28.2%)
Not specified	2 (4.3%)	9 (13.0%)^a^	9 (1.9%)
Flat size (m²)	88.0 ± 39.3 (median 80.0)^e^	93.9 ± 38.7 (median 90.0)	108.5 ± 46.3 (median 100.0)
per person	50.8 ± 29.5 (median 42.5)	48.0 ± 21.2 (median 47.5)	45.6 ± 24.6 (median 38.2)
Garden or balcony	44 (95.7%)	65 (94.2%)	463 (96.3%)
Severe physical health problem (e.g., diabetes, cancer, etc.)	6 (13.0%)^a,b^	2 (2.9%)	33 (6.9%)
Current psychiatric treatment	38 (82.6%)	53 (76.8%)	–
Current psychological/psychotherapeutic treatment	14 (30.4%)	31 (44.9%)	–

*SMI* serious mental illness; *MDD* major depressive disorder without psychotic features.

^a^Significantly (*p* < 0.05) higher compared to control group according to Fisher’s exact test (two-tailed).

^b^Significantly (*p* < 0.05) higher compared to MDD group according to Fisher’s exact test (two-tailed).

^c^Significantly (*p* < 0.05) lower compared to control group according to Fisher’s exact test (two-tailed).

^d^Significantly (*p* < 0.05) higher compared to control group according to Bonferroni-corrected Kruskal–Wallis test (two-tailed).

^e^Significantly (*p* < 0.05) lower compared to control group according to Bonferroni-corrected Kruskal–Wallis test (two-tailed).

At baseline, significantly more MDD patients than individuals suffering from SMI or controls were indecisive about the appropriateness of COVID-19-related public health policy measures. In addition, more MDD than SMI patients reported about an increased propensity to violence, while fewer SMI patients than MDD patients (T1) and controls (T1 + T2) felt burdened by the intensified presence of the police. From baseline to follow-up, the number of study participants who had conducted a SARS-CoV-2 test had increased significantly in all three groups. The number of subjects with a positive test results, in turn, had increased in the MDD and the control group, but not in the SMI group.

A variety of significant changes over time were detected in controls but not in the two patient groups. Next to an increased skepticism regarding the appropriateness of COVID-19-related public health policy measures and a decreased willingness to adhere to them, this included an increase in the proportion of study participants consuming alcohol or other substances in order to feel better and more people reporting about an increased propensity to violence. On the other hand, the number of control subjects feeling burdened by the intensified presence of the police decreased from baseline to follow-up (Table 2).

**Table 2:** COVID-19-related variables at baseline (T1) and follow-up (T2).

Variable		SMI (*N* = 46)	MDD (*N* = 69)	Controls (*N* = 481)
		*N* (%)	*N* (%)	*N* (%)
SARS-CoV-2 test				
Test conducted	T1 T2	19 (41.3%) 36 (78.3%)↑	23 (33.3%) 46 (71.0%)↑	164 (34.1%) 381 (79.2%)↑
Positive test result	T1 T2	1 (5.3%) 2 (4.3%)	0 (0.0%) 5 (10.2%)↑	11 (2.3%) 50 (13.1%)↑
Do you believe that the public health policy measures for the containment of the COVID-19 pandemic are appropriate?				
Rather yes/entirely yes	T1 T2	40 (87.0%) 39 (84.8%)	53 (76.8%) 51 (73.9%)	394 (81.9%) 354 (73.6%)↓
Neither nor	T1 T2	0 (0.0%)^a^ 3 (6.5%)	9 (13.0%)^b^ 3 (4.4%)	27 (5.6%) 34 (7.1%)
Rather not/not at all	T1 T2	6 (13.0%) 4 (8.7%)	7 (10.1%) 15 (21.7%)	60 (12.5%) 92 (19.2%)↑
Do you adhere to the recommended measures for the containment of the COVID-19 pandemic?				
Rather yes/entirely yes	T1 T2	43 (93.4%) 46 (100%)	61 (88.4%) 63 (91.3%)	468 (97.3%) 450 (93.6%)↓
Neither nor	T1 T2	1 (2.2%) 0 (0.0%)	0 (0.0%) 2 (2.9%)	4 (0.8%) 10 (2.1%)
Rather not/not at all	T1 T2	0 (0.0%) 0 (0.0%)	5 (7.6%) 2 (2.9%)	9 (1.9%) 9 (1.9%)
Did you consume alcohol or other substances since the outbreak of the COVID-19 pandemic in order to feel better?	T1 T2	5 (10.9%) 10 (21.7%)	16 (23.2%) 23 (33.3%)	84 (17.5%) 131 (27.3%)↑
Is the intensified presence of the police burdening you?	T1 T2	0 (0.0%)^a,c^ 2 (4.3%)^c^	11 (15.9%) 10 (14.5%)	119 (24.7%) 84 (17.5%)↓
Did/do you feel exposed to violence?	T1 T2	1 (2.2%) 0 (0.0%)	2 (2.9%) 3 (4.3%)	8 (1.7%) 11 (2.3%)
Has your propensity to violence increased?	T1 T2	1 (2.2%)^a^ 3 (6.5%)	11 (15.9%)^b^ 9 (13.0%)	33 (6.9%) 60 (12.5%)↑

↓ = Significant (*p* < 0.001) decrease in number at T2 compared to T1 according to McNemar-test.

↑ = Significant (*p* < 0.001) increase in number at T2 compared to T1 according to McNemar-test.

^a^Significantly (*p* < 0.05) lower compared to MDD group according to Fisher’s exact test (two-tailed).

^b^Significantly (*p* < 0.05) higher compared to control group according to Fisher’s exact test (two-tailed).

^c^Significantly (*p* < 0.05) lower compared to control group according to Fisher’s exact test (two-tailed).

### Psychological distress, resilience, perceived social support, loneliness, and boredom at baseline and follow-up

Table 3 provides a comparison of the three groups regarding psychological distress, resilience, perceived social support, loneliness, and boredom. In addition, Supplementary Table 1 provides means and standard deviations for the respective measures.

**Table 3:** Psychological distress, resilience, perceived social support, loneliness, and boredom at baseline (T1) and follow-up (T2).

Variable		SMI (*N* = 46)	MDD (*N* = 69)	Controls (*N* = 481)
*Psychological distress* (BSCL)		T value ≥ 63	T value ≥ 63	T value ≥ 63
		% (N)	% (N)	% (N)
Anger-hostility	T1	19.6% (9)	36.2%^b^ (25)	16.4% (79)
	*T2*	*21.7% (10)*	*33.3%*^*b*^ *(23)*	*17.5% (84)*
Anxiety	T1	30.4%^b^ (14)	47.8%^b^ (33)	16.0% (77)
	*T2*	*30.4%*^b^ *(14)*	*49.3%*^*a,b*^ *(34)*	*13.7% (66)*
Depression	T1	19.6% (9)	40.6%^a,b^ (28)	11.9% (57)
	*T2*	*15.2% (7)*	*40.6%*^*a,b*^*(28)*	*9.6% (46)*
Paranoid ideation	T1	10.9% (5)	29.0%^a,b^ (20)	10.6% (51)
	*T2*	*13.0% (6)*	*27.5%*^*b*^ *(19)*	*11.9% (57)*
Phobic anxiety	T1	43.5% (20)	50.7% (35)	41.0% (197)
	*T2*	*30.4% (14)*	*52.2%*^*b*^ *(36)*	*29.9%*↓** (144)
Psychoticism	T1	39.1%^b^ (18)	46.4%^b^ (32)	14.6% (70)
	*T2*	*23.9%*^b^ *(11)*	*42.0%*^*a,b*^ *(29)*	*13.3% (64)*
Somatization	T1	10.9% (5)	30.4%^a,b^ (21)	8.7% (42)
	*T2*	*10.9% (5)*	*33.3%*^*a,b*^ *(23)*	*7.9% (38)*
Interpersonal sensitivity	T1	23.9%^b^ (11)	40.6%^b^ (28)	9.1% (44)
	*T2*	*15.2% (7)*	*39.1%*^*a,b*^ *(27)*	*11.9% (57)*
Obsessive-compulsiveness	T1	26.1%^b^ (12)	43.5%^b^ (30)	12.1% (58)
	*T2*	*19.6% (9)*	*40.6%*^*a,b*^ *(28)*	*14.8% (71)*
Global Severity Index	T1	23.9% (11)	47.8%^a,b^ (33)	13.9% (67)
	*T2*	*23.9% (11)*	*44.9%*^*a,b*^*(31)*	*13.1% (63)*
*Resilience* (RS-13; range: 13–91)		% (N)	% (N)	% (N)
Low	T1	58.7%^b^ (27)	65.2%^b^ (45)	17.7% (85)
	*T2*	*50.0%*^*b*^ *(23)*	*53.6%*^*b*^ *(37)*↓*	*20.0% (96)*
Moderate	T1	15.2% (7)	14.5% (10)	19.3% (93)
	*T2*	*19.6% (9)*	*15.9% (11)*	*18.9% (91)*
High	T1	23.9%^c^ (11)	18.8%^c^ (13)	63.0% (303)
	*T2*	*30.4%*^*c*^ *(14)*	*30.4%*^*c*^ *(21)*↑*	*60.5% (291)*
*Perceived social support* (MSPSS; range: 1–5)		>50%	>50%	>50%
		% (N)	% (N)	% (N)
Total Score	T1	82.6%^c^ (38)	75.4%^c^ (52)	96.0% (462)
	*T2*	*93.5% (43)*	*87.0%*^*c*^ *(60)*	*95.4% (459)*
*Loneliness* (TILS; range: 3–9)		% (N)	% (N)	% (N)
Moderate (TILS score 5–6)	T1	43.5% (20)	37.7% (26)	29.7% (143)
	*T2*	*32.6% (15)*	*26.1%*^*c*^ *(18)*	*41.2% (198)*
Severe (TILS score ≥ 7)	T1	26.1% (12)	31.9% (22)	22.5% (108)
	*T2*	*30.4% (14)*	*31.9%*^*b*^ *(22)*	*18.5% (89)*
*Boredom* (MSBS-SF; range: 8–56)		Mean ± SD	Mean ± SD	Mean ± SD
	T1	27.1 ± 9.1	29.4 ± 12.4^d^	25.6 ± 10.6
	*T2*	*24.8* *±* *9.6*	*29.2* *±* *11.7*^*d*^	*25.4* *±* *11.5*

For clarity reason: italic formatting shows the second time of examination.

*SMI* serious mental illness, *MDD* major depressive disorder without psychotic features, *BSCL* Brief Symptom Checklist, *RS-13* Resilience Scale (short form), *MSPSS* Multidimensional Scale of Perceived Social Support, *TILS* Three-Item Loneliness Scale, *MSBS-SF* Multidimensional State Boredom Scale-Short Form.

↓* = significant (*p* < 0.05) decrease between T1 and T2 according to McNemar-test.

↑* = significant (*p* < 0.05) increase between T1 and T2 according to McNemar-test.

↓** = significant (*p* < 0.001) decrease between T1 and T2 according to McNemar-test.

^a^Significantly (*p* < 0.05) higher compared to SMI group according to Fisher’s exact test (two-tailed).

^b^Significantly (*p* < 0.05) higher compared to control group according to Fisher’s exact test (two-tailed).

^c^Significantly (*p* < 0.05) lower compared to control group according to Fisher’s exact test (two-tailed).

^d^Significantly (*p* < 0.05) higher compared to control group according to Bonferroni-corrected ANOVA.

Concerning psychological distress, significantly more MDD patients than SMI patients or controls achieved BSCL *T*-scores ≥ 63, thus suffering from clinically relevant psychological symptoms. This was true for most BSCL subscales as well as the GSI at both assessment times. With the exception of a higher prevalence of clinically relevant interpersonal sensitivity and obsessive-compulsiveness at baseline as well as anxiety and psychoticism at both assessment times, SMI patients, in turn, were largely comparable with controls in this regard. A significant decrease of symptoms from baseline to follow-up was solely observed in controls and concerned the BSCL subscale ‘phobic anxiety’.

Compared to controls, SMI patients achieved significantly higher mean scores in four out of nine BSCL subscales (T1: depression, interpersonal sensitivity, obsessive-compulsiveness; T1 + T2: psychoticism) and the GSI, whereas MDD patients scored significantly higher in all subscales and the GSI at both assessment times. Comparing the two patient samples in this regard, a significant difference was found in the GSI and all BSCL subscales except ‘anger-hostility’ (higher scores in MDD patients).

At both assessment times, mean RS-13 scores and the number of cases indicating high resilience were significantly lower in both patient groups compared to controls. In turn, significantly more SMI and MDD patients than controls indicated low resilience. SMI patients achieved significantly higher RS-13 mean scores at baseline compared to MDD patients, however, this difference vanished at follow-up. Only MDD patients indicated a significant increase of resilience over time (increase in RS-13 mean score; decrease in the low/increase in the high resilience category).

MSPSS mean scores were generally significantly higher in controls than in the two patient groups. This concerned perceived social support from family, from friends, and from other significant close relationships. At baseline, high perceived social support was significantly more frequently detected in controls compared to the two patient groups. In spite of a significant increase in the MSPSS mean total score over time, MDD patients still reported high perceived social support significantly less frequently than controls at follow-up, whereas the percentage of SMI patients and controls with high perceived social support was comparable at T2.

The three groups were comparable regarding the number of study participants experiencing moderate or severe loneliness at baseline. At follow-up, the number of cases with moderate loneliness decreased in both patient groups and increased in the control group, however, these within-group changes did not attain statistical significance. The prevalence of severe loneliness, in turn, increased in SMI patients (not significant), remained unchanged in MDD patients, and decreased in controls (not significant). Altogether, at T2, a significantly lower prevalence of moderate loneliness and a significantly higher prevalence of severe loneliness was detected in MDD patients compared to controls.

At both assessment times, the degree of boredom was comparable between SMI patients and controls and was significantly higher in MDD patients than in controls.

### Associations of psychological distress with sociodemographic variables, resilience, perceived social support, loneliness, and boredom

Tables 4 and 5 show the correlations between BSCL subscales at baseline/follow-up (Table 4) or BSCL difference scores between T1 and T2 (Table 5) and sociodemographic variables as well as the covariates at baseline measurement (Table 4/Table 5) and covariates difference scores between T1 and T2 (Table 5).

**Table 4:** Correlations between T1 and T2 variables (Spearman’s rho).

BSCL		Age	Sex (female)	Residence (Austria)	RS-13 T1	MSPSS T1	TILS T1	MSBS-SF T1
Anger-hostility	T1	−0.178**	0.142**	−0.058	−0.333**	−0.262**	0.412**	0.501**
	*T2*	−0.158**	0.177**	*−0.073*	−0.363**	−0.214**	0.348**	0.424**
Anxiety	T1	−0.071	0.131*	−0.101*	−0.391**	−0.311**	0.447**	0.519**
	*T2*	−0.095*	0.133**	−0.093*	−0.485**	−0.328**	0.384**	0.489**
Depression	T1	−0.139**	0.093*	−0.057	−0.497**	−0.361**	0.604**	0.642**
	*T2*	−0.086*	0.106**	*−0.065*	−0.527**	−0.424**	0.490**	0.545**
Paranoid ideation	T1	−0.061	0.060**	−0.040	−0.382**	−0.442**	0.430**	0.409**
	*T2*	−0.103*	*0.080*	*−0.004*	−0.380**	−0.361**	0.352**	0.345**
Phobic anxiety	T1	−0.044	0.086*	−0.045	−0.285**	−0.185**	0.283**	0.256**
	*T2*	*−0.076*	0.123**	*0.004*	−0.367**	−0.251**	0.303**	0.317**
Psychoticisim	T1	−0.058	0.050	−0.038	−0.456**	−0.433**	0.525**	0.490**
	*T2*	*−0.024*	*0.059*	*−0.006*	−0.496**	−0.425**	0.442**	0.442**
Somatization	T1	0.041	0.159**	−0.075	−0.311**	−0.291**	0.314**	0.407**
	*T2*	*0.006*	0.201**	*−0.039*	−0.391**	−0.265**	0.272**	0.341**
Interpersonal sensitivity	T1	−0.105*	0.126**	−0.076	−0.448**	−0.391**	0.516**	0.494**
	*T2*	−0.093*	0.131**	*0.020*	−0.523**	−0.388**	0.463**	0.441**
Obsessive compulsiveness	T1	−0.112**	0.071	−0.004	−0.494**	−0.341**	0.449**	0.602**
	*T2*	−0.121**	0.111**	*0.025*	−0.539**	−0.318**	0.401**	0.487**
Global Severity Index	T1	−0.108**	0.120**	−0.066	−0.504**	−0.410**	0.566**	0.618**
	*T2*	−0.113**	0.154**	*−0.028*	−0.553**	−0.399**	0.464**	0.516**

For clarity reason: italic formatting shows the second time of examination.

*p < 0.05; **p < 0.01.

**Table 5:** Correlations of difference scores between T1 and T2 (Spearman’s rho).

BSCL	Age T1	Sex (female) T1	Residence (Austria) T1	RS-13 T1	MSPSS T1	TILS T1	MSBS-SF T1	RS-13 diff.	MSPSS diff.	TILS diff.	MSBS-SF diff.
Anger-hostility diff.	0.008	0.032	0.012	−0.057	0.019	−0.065	−0.085*	−0.082*	−0.079	0.144**	0.209**
Anxiety diff.	−0.068	−0.030	0.029	−0.054	0.041	−0.102*	−0.075	−0.093*	−0.165**	0.210**	0.165**
Depression diff.	0.050	0.009	−0.001	0.003	−0.002	−0.171**	−0.159**	−0.180**	−0.168**	0.301**	0.322**
Paranoid ideation diff.	−0.071	0.027	0.029	0.028	0.095	−0.080	−0.059	−0.200**	−0.165**	0.136**	0.140**
Phobic anxiety diff.	−0.033	0.030	0.032	−0.027	−0.046	−0.024	0.019	−0.072	−0.014	0.133**	0.064
Psychoticism diff.	0.003	0.013	0.054	0.017	0.028	−0.113*	−0.075	−0.119**	−0.072	0.156**	0.109**
Somatization diff.	−0.028	0.049	0.047	−0.087	−0.022	−0.035	−0.054	−0.034	−0.070	0.116**	0.087*
Interpersonal sensitivity diff.	−0.047	0.035	0.062	−0.075	0.030	−0.042	−0.021	−0.158**	−0.148**	0.174**	0.182**
Obsessive compulsiveness diff.	−0.038	0.033	0.013	−0.026	0.033	−0.089*	−0.146**	−0.150**	−0.086*	0.218**	0.271**
Global Severity Index diff.	−0.050	0.053	0.051	−0.039	0.034	−0.138**	−0.161**	−0.183**	−0.116**	0.264**	0.258**

*BSCL* Brief Symptom Checklist, *RS-13* Resilience Scale (short form), *MSPSS* Multidimensional Scale of Perceived Social Support, *TILS* Three-Item Loneliness Scale, *MSBS-SF* Multidimensional State Boredom Scale-Short Form, *diff.* difference.

*p < 0.05; **p < 0.01.

Female sex and younger age correlated weakly with most BSCL subscales at T1 and T2, whereas ‘anxiety’ was the only subscale to be weakly related to residence at both assessment times (higher scores in Italian study participants). There was no significant relationship between BSCL difference scores between T1 and T2 and age, sex, or residence.

As expected, the severity of psychological symptoms (BSCL) was generally negatively associated with resilience and perceived social support, and positively associated with loneliness and boredom. The same was true concerning the relationship between changes in BSCL subscales and changes in covariates over time. At baseline, highest correlations were detected between depression and both boredom (*r* = 0.642) and loneliness (*r* = 0.604), whereas at follow-up, highest correlations were detected between the GSI and resilience (*r* = −0.553), and between depression (*r* = −0.424) and psychoticism (*r* = −0.425) and perceived social support. Concerning changes in correlations between GSI scores at T1 and T2 with the scores of covariates, a significant decrease was only found for loneliness (*z* = 2.397, *p*_fisher_ = 0.017) and boredom (*z* = 2.589, *p*_fisher_ = 0.010).

Lowest correlations within the respective measure were found with phobic anxiety at T1 (resilience: *r* = −0.285; perceived social support: *r* = −0.185; loneliness: *r* = 0.283; boredom: *r* = 0.256). Notably, in the long-term, the highest correlations were detected between changes in depression and changes in both boredom (*r* = 0.322) and loneliness (*r* = 0.301).

Repeated measures analyses of covariance revealed significant GSI score differences between SMI patients, MDD patients, and controls (*F*[2, 578] = 8.655, *p* < 0.001, η_p_^2^ = 0.029) with MDD patients showing higher GSI scores compared to the other groups. Furthermore, age, sex, resilience, perceived social support, loneliness, and boredom were significant predictors of the GSI score (Table 5).

As shown in Supplementary Table 2, the same variables as described above occurred as significant predictors for the GSI, except for age, which was only significant at T2.

Regarding within-subjects effects, an interaction between time and age was significant, meaning that younger study participants reported higher GSI scores compared to older participants at follow-up, which was not significant at baseline. The interactions between time and loneliness on one hand and between time and boredom on the other indicate that loneliness and boredom were stronger predictors of the GSI at baseline than at follow-up (Table 6). Regarding the results of the analyses accounting for time of assessment and COVID-19 incidence rate, neither of the two variables were significant explanatory factors. This applied for both Tyrol and South Tyrol. Moreover, the results of the repeated measures ANCOVA above remained unchanged (see Supplementary Table 3).

**Table 6:** Repeated Measures ANCOVA (z-standardized).

		SS	df	MS	*F*	partial η^2^	*p*
*GSI score*	*Between-subjects effects*						
	Group (SMI vs. MDD vs. controls)	3.937	2	1.969	8.655	0.029	<0.001
	Age	0.937	1	0.937	4.122	0.007	0.043
	Sex (female vs. male)	2.218	1	2.218	9.751	0.017	0.002
	Residence (Austria vs. Italy)	0.002	1	0.002	0.008	<0.001	0.930
	RS-13 T1	14.305	1	14.305	62.891	0.098	<0.001
	MSPSS T1	7.569	1	7.569	33.275	0.054	<0.001
	TILS T1	10.437	1	10.437	45.884	0.074	<0.001
	MSBS-SF T1	16.290	1	16.290	71.614	0.110	<0.001
	*Within-subjects effects*						
	Time	0.047	1	0.047	0.804	0.001	0.370
	Time × Group (SMI vs. MDD vs. controls)	0.091	2	0.046	0.781	0.003	0.459
	Time × Sex (female vs. male)	0.161	1	0.161	2.753	0.005	0.098
	Time × Age	0.278	1	0.278	4.769	0.008	0.029
	Time × Residence (Austria vs. Italy)	0.026	1	0.026	0.443	0.001	0.506
	Time × RS-13 T1	0.039	1	0.039	0.666	0.001	0.415
	Time × MSPSS T1	0.080	1	0.080	1.366	0.002	0.243
	Time × TILS T1	0.633	1	0.633	10.853	0.018	0.001
	Time × MSBS-SF T1	0.302	1	0.302	5.170	0.009	0.023

		Estimated			95% CI		
		Mean	S.E.		LB	UB	N
	SMI	0.47	0.053		0.365	0.575	42
	MDD^a,b^	0.70	0.047		0.607	0.790	65
	Controls	0.50	0.016		0.469	0.531	481

*SMI* serious mental illness, *MDD* major depressive disorder without psychotic features, *BSCL* Brief Symptom Checklist, *RS-13* Resilience Scale (short form), *MSPSS* Multidimensional Scale of Perceived Social Support, *TILS* Three-Item Loneliness Scale, *MSBS-SF* Multidimensional State Boredom Scale-Short Form, *SS* sum of squares, *df* degree of freedom, *MS* mean square, *LB* lower bound, *UB* upper bound.

^a^Significantly different from SMI group (*p* < 0.001; estimated marginal mean difference).

^b^Significantly different from control group (*p* < 0.001; estimated marginal mean difference).

## Discussion

The current longitudinal study investigated mental health in individuals with pre-existing SMI or MDD and people with no self-reported mental health disorders from Tyrol (Austria) and South Tyrol (Italy) and the related impact of both protective and risk factors during the COVID-19 pandemic. Overall, we found evidence for a particular burden in people with MDD both at baseline and at five-month follow-up, i.e., levels of psychological symptoms as assessed by the BSCL and the prevalence of clinically relevant psychological distress as defined by the GSI were significantly higher in this group compared to the other two groups. SMI patients and controls, in turn, differed mainly in terms of psychoticism. Notably, the prevalence of clinically relevant psychological symptoms remained virtually unchanged among each group over time and a lower degree of both resilience and perceived social support as well as loneliness and boredom significantly predicted psychological distress.

The prevalence of psychological distress was comparable between study participants from Tyrol and South Tyrol, however, Italian study participants were more prone to anxiety than those residing in Austria. In view of considerably higher numbers of SARS-CoV-2 cases and COVID-19-related deaths and substantially stricter public health policy measures in Italy, this is not surprising.

Women, younger individuals, and people with pre-existing mental health conditions presented with particularly high levels of psychological distress, which corroborates the results of previous studies from different countries^6,7,9,22–27^. In line with the findings of a recent study from the United States^28^, 13.9% of control subjects (67/481) reported psychological distress at baseline relative to 13.1% at follow-up. Importantly, this prevalence was almost twice as high in SMI patients (not significant) and significantly higher in patients suffering from MDD (47.8% [33/69] and 44.9%, respectively). This finding points to a considerable long-term impact of the current situation on both people suffering from SMI and MDD, however, it also indicates that the subjective perception of mental distress of individuals with SMI was more equal to that of people with no self-reported mental health disorders than that of individuals with MDD. Comparing the two patient samples in this regard, individuals with MDD again achieved significantly higher scores in the GSI and most BSCL subscales. As previously shown by Hao et al.^8^, our results therefore point to a particularly negative impact of the pandemic on non-psychotic individuals with a pre-existing psychiatric disorder. On the other hand and following Muruganandam et al.^4^, one could also assume a particular lack of COVID-19 knowledge among individuals suffering from SMI. This issue cannot be addressed by our data.

As suggested by Hölzle et al.^16^, one could speculate that SMI patients might be occupied with serious intrinsic issues and might therefore be pseudo-resilient toward adverse situational or behavioral changes, e.g., in the context of a pandemic. Theoretically, this could also explain why the intensified presence of the police was burdening MDD patients and controls, but not SMI patients. However, while both patient groups consistently indicated a lower degree of resilience compared to controls, which is in line with the findings of pre-pandemic studies^29,30^, RS-13 mean scores of individuals with SMI and MDD differed only at baseline (lower scores in MDD patients), but not at follow-up. Similarly, the two patient groups were largely comparable regarding the prevalence of high perceived social support, loneliness, and boredom over time. Notwithstanding, psychological symptoms as assessed by the BSCL were consistently more pronounced in people with MDD. Our results therefore suggest that patients suffering from MDD may be likely to perceive the COVID-19 pandemic and related public health policy measures as particularly distressing, in line with the findings of Solé et al.^10^. One has to consider, however, that baseline assessments were done in the second half of 2020, i.e., after the onset of the pandemic, and causal relationships can thus not be drawn from our data. In addition, due to the design of the survey, we could not assess psychopathological symptoms and could therefore not determine whether the two patient groups differed in terms of symptomatology at the time of study conduction, which clearly could have affected our results.

Next to resilience, the level of perceived social support was consistently significantly lower in both patient groups compared to controls, whereas the prevalence and degree of loneliness was comparable between the three groups at baseline and significantly higher in MDD patients than in controls at follow-up. In addition, throughout the course of the study, individuals suffering from MDD felt more bored than those with SMI (not significant) and those with no self-reported mental health disorders. Generally, loneliness has been one of the most frequently identified personal stress factors during the COVID-19 pandemic^31^ and similarly, boredom has previously been shown to correlate with the occurrence of depression, anxiety, and stress^32^. Accordingly, our findings suggest that individuals with MDD may be highly vulnerable and thus in particular need of support in the context of the pandemic, at least in comparison to those with no self-reported mental health disorders and, to a lesser extent, those with SMI. This issue clearly warrants additional investigation.

Overall, loneliness and boredom were stronger predictors of psychological distress at baseline than at follow-up. On the one hand, this may indicate that study participants were able to cope with these risk factors by changing their behavior in a positive sense and reaching a more satisfying situation over time. However, our finding of an increased propensity to violence and maladaptive strategies like an increased consumption of alcohol or other substances in control subjects over time is highly alarming. Similarly, significantly higher GSI scores in younger study participants compared to older participants at follow-up, i.e., a deterioration of psychological well-being over time, is a matter of major concern. These results emphasize the need of specific prevention and mitigation strategies to address these public health problems. Obviously, they have to be tailored to the special needs of various groups.

The present study has several limitations. First, all results came from a voluntary online survey, were based on self-reported data, and may thus be biased. Also, the response rate was rather low. Second, diagnoses of study participants with severe mental disorders were gathered from chart information and there were no standardized assessments of psychopathology at the time of the study. Similarly, control subjects had to self-report not suffering from mental health disorders. This clearly limits generalizability to the whole population of Tyrol and South Tyrol. In addition, as mentioned above, we do not have pre-pandemic baseline measures and can thus not be sure that the high levels of psychological distress, loneliness, and boredom among our sample are merely a result of the pandemic. Third, at baseline, the questions/scales were specifically phrased to enquire about the period of time when COVID-19-related public health policy measures were most pronounced, however, recall bias has to be taken into account. Lastly, one has to consider some major demographic and functional differences between study participants with severe mental disorders and control subjects. For example, more than 80% of controls were in a relationship and were therefore at least able to rely on each other for support, whereas the SMI and MDD groups were far lower in terms of their relationship status. Similarly, patients and controls differed significantly in terms of their work and income status. These findings had to be expected and could easily have affected some of the results, such as loneliness or boredom.

Despite these limitations, our findings suggest a negative long-lasting impact of the pandemic on mental health of people both with and without mental disorders. Accordingly, there is the urgent need for governments to have policies in place to alleviate the potential threat of COVID-19 on the mental health of the population, and targeted interventions focusing on the specific needs of various groups (including those with no mental health disorders) are essential. For example, resilience-trainings focussing on mindfulness and cognitive behavioral skills^33^, physical exercise programs with a focus on outdoor activity^34^, or interventions such as supportive text message programs (e.g., Text4Hope^35^, launched in Alberta, Canada) may reduce psychological distress and loneliness, and increase well-being. As the COVID-19 pandemic continues and infection rates have worsened subsequent to our follow-up survey, it will be critical to understand how mental health may further change. We are therefore continuing to conduct follow-up surveys in the same participants.

## Methods

### Participants

Local people who had been admitted to a psychiatric ward and had been diagnosed with a mental health disorder in 2019 as well as a control group from the general population aged 18 and above were invited to complete an online survey. Altogether, 1542 patients diagnosed with SMI^18^ and 1054 patients with MDD were invited by mail to participate, of which 190 enrolled. Diagnoses were confirmed using chart information. 46 out of 99 individuals in the SMI group (SSD: *n* = 14, BD: *n* = 23, MDD with psychotic features: *n* = 9) and 69 out of 91 individuals in the MDD group completed both baseline and follow-up surveys and were included in the analyses of the current report. The control group was recruited through advertising in print media, email lists, flyer, and social media. A total of 1646 people participated in the baseline survey (findings obtained in the Tyrolean sub-sample are reported in Tutzer et al.^22^ and Chernova et al.^36^). They were asked to provide an email address in order to be reminded for follow-up, however, this was not a prerequisite to participate in the baseline survey. All participants provided informed consent online. Control subjects reporting to have been diagnosed with a mental health disorder in the past as well as those who reported on current psychopharmacological and/or psychotherapeutic treatment were excluded from the analyses. Of the final sample (*N* = 1197), 481 individuals completed both baseline and follow-up surveys and are considered in this report. At the end of the survey, all participants received a downloadable information sheet on professional support numbers and addresses.

### Procedures

In Tyrol, survey responses were collected between June 26th, 2020 and September 13th, 2020 (T1, baseline) and between November 30th, 2020 and January 24th, 2021 (T2, follow-up). For organizational reasons, data collection took place at a later date in South Tyrol (baseline between September 7th, 2020 and November 22nd, 2020; follow-up between February 8th, 2021 and April 4th, 2021), however, the time interval between surveys was equal in both countries. Electronic data capture was conducted by means of the Computer-based Health Evaluation System (CHES)^37^. Next to the registration of sociodemographic and COVID-19-related aspects (e.g., whether participants considered the public health policy measures to be appropriate, whether they felt exposed to violence, or whether their propensity to violence had increased) standardized questionnaires were used. During baseline assessment, participants were asked to refer to the period of time when COVID-19-related public health policy measures were most pronounced, whereas the follow-up assessment related to the current circumstances.

The subjective perception of mental distress was assessed using the self-rated Brief Symptom Checklist (BSCL)^38^. It is composed of 53 items to be answered on a five-point Likert-type scale (0–4), ranging from “not at all/no distress” to “extremely/very strong distress”. Psychological distress is reflected in nine subscales: anger-hostility, anxiety, depression, paranoid ideation, phobic anxiety, psychoticism, somatization, interpersonal sensitivity, and obsessive-compulsiveness. BSCL raw values were transformed into age- and gender-specific normative T-scores (range: 50 ± 10; higher values indicate greater psychological distress) using a standardization reference table. In addition, the Global Severity Index (GSI), a composite score of the BSCL used to measure general distress, was calculated (T-scores ranging from 20 = lowest to 80 = worst psychological distress). Cases with clinically relevant psychological symptoms were defined according to T-criteria. A T-score ≥63 was defined to indicate subjective impairment.

Resilience was evaluated using the 13-item short form of the Resilience Scale (RS-13)^39^. All items are scored on a seven-point scale, ranging from 1 = “strongly disagree” to 7 = “strongly agree” with possible scores ranging from 13 to 91. The higher the total score, the higher is resilience. The overall RS-13 score is categorized into three levels: scores below 66 reflect low resilience, scores between 67 and 72 indicate a moderate level of resilience, and scores of 73 and higher indicate high resilience.

Perceived social support was assessed using the Multidimensional Scale of Perceived Social Support (MSPSS)^40^, a 12-item, self-report scale that examines social support on three subscales: family, friends, and significant others. Items were scored on a five-point Likert scale, ranging from 1 = “strongly disagree” to 5 = “strongly agree”. Scores >50% indicate high perceived support.

Loneliness was measured by using the short form of the Revised University of California Los Angeles Loneliness Scale, the Three-Item Loneliness Scale (TILS)^41^. The TILS total score ranges from 3 to 9 with higher scores indicating a higher degree of loneliness. Scores ≥7 indicate severe loneliness, whereas a score of 5 or 6 indicates moderate loneliness ^42^.

Boredom was assessed using the Multidimensional State Boredom Scale-Short Form (MSBS-SF)^43^, which consists of eight Likert-type items that are rated on a seven-point scale (1 = “strongly disagree”, 7 = “strongly agree”), yielding a maximum score of 56. Higher scores indicate a higher degree of boredom.

### Statistical analysis

Statistical analyses were conducted with IBM SPSS 27^44^. Sociodemographic data, COVID-19-related aspects, critical T values, and means of outcome variables within the SMI, MDD, and control groups were compared by nonparametric test procedures for dichotomous, categorical, and metric variables (Fisher’s exact test, Kruskal–Wallis test, and ANOVA [Bonferroni-corrected]) at baseline. For baseline and follow-up comparison, the McNemar-test was conducted. Associations between BSCL subscales and age, sex, residence, and covariates (RS-13, MSPSS, TILS, MSBS-SF) for T1 and T2 as well as for difference scores were investigated by means of Spearman rank correlations. According to Cohen^45^, correlation coefficients can be interpreted as follows: *r* < 0.10 no correlation; *r* = 0.10–0.29 low correlation; *r* = 0.30–0.49 moderate correlation, and *r* ≥ 0.50 high correlation. Repeated measures ANCOVA was used in order to account for main effects and interactions of covariates and multiple assessments on GSI. The three groups (SMI, MDD, controls) were used as factor, time as variable of multiple assessments (T1 and T2), and age, sex, residence, and questionnaire scores (RS-13, MSPSS, TILS, MSBS-SF) as covariates. Effect sizes expressed by partial η^2^ can be interpreted as: η_p_^2^ ≥ 0.01 small effect; η_p_^2^ ≥ 0.06 medium effect, and η_p_^2^ ≥ 0.14 large effect. Since assessment periods between Tyrol and South Tyrol differed at baseline and follow-up, first-order autoregressive linear mixed model analyses were used to account for both the time of assessment and the COVID-19 7-day incidence rate. These two variables were included in addition to the variables in the repeated measures ANCOVA described above. The date of participation was transformed into the respective month and was used as a factor. The COVID-19 7-day incidence rate at the respective day of participation in the corresponding region was included as a covariate.

### Ethical approval

In Austria, the study was approved by the Ethics Committee of the Medical University Innsbruck. In Italy, the project was approved by the Ethics Committee of the Sanitary Agency of South Tyrol.

### Reporting summary

Further information on research design is available in the Nature Research Reporting Summary linked to this article.

## Supplementary information

Supplementary Information

REPORTING SUMMARY

## Data Availability

According to the Austrian and Italian law, data sharing requires approvals from the regional Committees for Medical and Health Research Ethics and from the regional Data Protection Officers. The data are therefore not publicly available. The data that support these findings can be provided by A.H., Medical University Innsbruck, upon reasonable request.
