# Supplementary material for: Mental health in individuals with severe mental disorders during the covid-19 pandemic: a longitudinal investigation
Source: Schizophrenia (Heidelb). 2022 Mar 8;8(1):17. doi: 10.1038/s41537-022-00225-z (PMC8903129; doi:10.1038/s41537-022-00225-z)
Supplement: Supplementary file 1 — Supplementary Information [file 41537_2022_225_MOESM1_ESM.pdf]

Supplementary Table 1: Mean and standard deviation of psychological distress, resilience, and perceived social support at baseline (T1) and follow-up (T2)

Variable		SMI (N=46)	MDD (N=69)	Controls (N=481)
**Psychological distress** (BSCL)		Mean ± SD	Mean ± SD	Mean ± SD
Anger-hostility	T1	0.58 ± 0.64	0.85 ± 0.86[a]	0.47 ± 0.55
	T2	0.54 ± 0.57	0.82 ± 0.86[a]	0.50 ± 0.57
Anxiety	T1	0.48 ± 0.53[d]	1.15 ± 1.06[a]	0.43 ± 0.56
	T2	0.56 ± 0.63[d]	1.08 ± 0.99[a]	0.41 ± 0.55
Depression	T1	0.80 ± 0.74[a,d]	1.31 ± 1.16[a]	0.51 ± 0.65
	T2	0.68 ± 0.63[d]	1.22 ± 1.07[a]	0.50 ± 0.62
Paranoid ideation	T1	0.67 ± 0.62[d]	1.07 ± 1.00[a]	0.50 ± 0.61
	T2	0.67 ± 0.75	0.97 ± 0.90[a]	0.55 ± 0.67
Phobic anxiety	T1	0.61 ± 0.72[d]	1.00 ± 1.08[a]	0.54 ± 0.56
	T2	0.45 ± 0.58[d]	0.89 ± 1.07[a]	0.43 ± 0.56 ↓
Psychoticism	T1	0.50 ± 0.49[a,d]	0.91 ± 0.86[a]	0.28 ± 0.44
	T2	0.50 ± 0.53[a,d]	0.79 ± 0.79[a]	0.27 ± 0.45
Somatization	T1	0.43 ± 0.62[d]	0.75 ± 0.84[a]	0.28 ± 0.47
	T2	0.37 ± 0.49[d]	0.78 ± 0.79[a]	0.31 ± 0.49
Interpersonal sensitivity	T1	0.80 ± 0.67[a,d]	1.22 ± 1.08[a]	0.47 ± 0.56
	T2	0.78 ± 0.81[d]	1.14 ± 1.01[a]	0.55 ± 0.65 ↑
Obsessive-compulsiveness	T1	0.87 ± 0.71[a,d]	1.32 ± 1.09[a]	0.52 ± 0.60
	T2	0.83 ± 0.58[d]	1.19 ± 0.94[a]	0.60 ± 0.63 ↑
Global Severity Index	T1	0.63 ± 0.51[a,d]	1.06 ± 0.89[a]	0.44 ± 0.45
	T2	0.59 ± 0.50[d]	0.99 ± 0.78[a]	0.45 ± 0.48
**Resilience** (RS-13; range: 13-91)				
	T1	63.1 ± 12.4[b,c]	57.6 ± 17.5[b]	73.6 ± 9.8
	T2	*65.7 ± 11.9[b]*	*60.1 ± 16.9[b]* ↑	*72.7 ± 11.6*
**Perceived social support** (MSPSS; range: 1-5)				
Total Score	T1	3.93 ± 0.85[b,c]	3.58 ± 0.92[b]	4.38 ± 0.62
	T2	*4.03 ± 0.64[b]*	*3.80 ± 0.88[b]* ↑	*4.38 ± 0.63*
Family	T1	3.83 ± 1.17[b]	3.51 ± 1.14[b]	4.24 ± 0.85
	T2	*3.99 ± 0.73[b]*	*3.61 ± 1.20[b]*	*4.28 ± 0.80*
Friends	T1	3.65 ± 1.15[b]	3.31 ± 1.14[b]	4.30 ± 0.77
	T2	*3.75 ± 1.00[b]*	*3.66 ± 1.07[b]* ↑	*4.28 ± 0.80*
Significant Other	T1	4.32 ± 0.80[b,c]	3.92 ± 1.07[b]	4.59 ± 0.60
	T2	*4.40 ± 0.55*	*4.13 ± 0.95[b]*	*4.60 ± 0.60*

Abbreviations: SMI=serious mental illness; MDD=major depressive disorder without psychotic features; BSCL=Brief Symptom Checklist; RS-13=Resilience Scale (short form); MSPSS=Multidimensional Scale of Perceived Social Support.

[a] significantly (p<.05) higher compared to control group according to Bonferroni-corrected ANOVA.

[b] significantly (p<.05) lower compared to control group according to Bonferroni-corrected ANOVA.

[c] significantly (p<.05) higher compared to MDD group according to Bonferroni-corrected ANOVA.

[d] significantly (p<.05) lower compared to MDD group according to Bonferroni-corrected ANOVA.

↑ significant (p <.05) increase between T1 and T2 according to Bonferroni-corrected pairwise T-Test.

↓ significant (p <.05) decrease between T1 and T2 according to Bonferroni-corrected pairwise T-Test.

Supplementary Table 2: Parameter estimates of GSI at T1 and T2 with repeated measures ANCOVA (z-standardized)

		Standardized coeff.		t	95% CI		partial η^2	*p*
		β	S.E.		LB	UB		
**GSI (T1)**	SMI vs. controls (Group)	-0.023	0.060	-0.380	-0.141	0.095	<.001	.704
	MDD vs. controls (Group)	0.231	0.054	4.286	0.125	0.337	.031	<.001
	MDD vs. SMI (Group)	0.254	0.072	3.539	0.113	0.395	.021	<.001
	Age	-0.014	0.016	-0.869	-0.045	0.017	.001	.385
	Sex (female vs. male)	0.033	0.015	2.147	0.003	0.063	.008	.032
	Residence (Austria vs. Italy)	-0.012	0.030	-0.400	-0.035	0.023	<.001	.689
	RS-13 T1	-0.136	0.019	-7.070	-0.174	-0.098	.080	<.001
	MSPSS T1	-0.086	0.018	-4.869	-0.121	-0.051	.039	<.001
	TILS T1	0.230	0.029	7.940	0.105	0.174	.098	<.001
	MSBS-SF T1	0.165	0.018	9.042	0.129	0.201	.124	<.001
**GSI (T2)**	SMI vs. controls (Group)	-0.036	0.066	-0.542	-0.167	0.095	.001	.588
	MDD vs. controls (Group)	0.167	0.060	2.799	0.050	0.284	.013	.005
	MDD vs. SMI (Group)	0.203	0.079	2.557	0.047	0.359	.011	.011
	Age	-0.047	0.017	-2.667	-0.008	-0.081	.012	.008
	Sex (female vs. male)	0.057	0.017	3.370	0.024	0.090	.019	.001
	Residence (Austria vs. Italy)	0.004	0.017	0.212	-0.029	0.036	<.001	.832
	RS-13 T1	-0.151	0.021	-7.096	-0.193	-0.109	.080	<.001
	MSPSS T1	-0.106	0.020	-5.409	-0.144	-0.067	.048	<.001
	TILS T1	0.085	0.019	4.342	0.046	0.123	.032	<.001
	MSBS-SF T1	0.125	0.020	6.218	0.086	0.165	.063	<.001

Abbreviations: MSBS-SF=Multidimensional State Boredom Scale-Short Form; RS-13=Resilience Scale (short form); MSPSS=Multidimensional Scale of Perceived Social Support; TILS=Three-Item Loneliness Scale; S.E. = standard error; LB = lower bound; UB = upper bound

Supplementary Table 3: Estimates of fixed effects in the Linear Mixed Model with AR(1) covariance structure including GSI as dependent variable and z-standardized covariates

		Standardized coeff.		t	95% CI		*p*
		β	S.E.		LB	UB	
**Factors**	SMI vs. controls (Group)	0.063	0.054	1.156	-0.044	0.170	.248
	MDD vs. controls (Group)	0.305	0.047	6.513	0.213	0.397	<.001
	MDD vs. SMI (Group)	0.242	0.063	3.834	0.118	0.366	<.001
	MoP – July 2020	0.955	2.590	0.369	-4.130	6.040	.713
	MoP – August 2020	1.011	1.568	0.644	-2.068	4.089	.519
	MoP – September 2020	1.883	1.392	1.353	-0.849	4.616	.176
	MoP – October 2020	0.199	0.971	0.205	-1.707	2.105	.837
	MoP – November 2020	0.691	0.985	0.701	-1.244	2.625	.484
	MoP – December 2020	0.328	0.969	0.339	-1.574	2.230	.735
	MoP – January 2021	-0.176	1.655	-0.106	-3.425	3.072	.915
	MoP – February 2021	0.047	1.052	0.045	-2.017	2.112	.964
	MoP – March 2021	0.220	0.977	0.225	-1.697	2.137	.822
	MoP – April 2021 (reference)	0[a]	0[a]	-	-	-	-
**Covariates**	Age	-0.036	0.014	-2.535	-0.063	-0.008	.012
	Sex (female vs. male)	0.052	0.014	3.802	0.025	0.079	<.001
	Residence (Austria vs. Italy)	0.250	0.585	0.428	-0.899	1.399	.669
	RS-13	-0.108	0.013	-8.241	-0.133	-0.082	<.001
	MSPSS	-0.110	0.013	-8.150	-0.137	-0.084	<.001
	TILS	0.111	0.012	9.335	0.088	0.135	<.001
	MSBS-SF	0.136	0.013	10.753	0.111	0.160	<.001
	COVID-19 IR	0.361	1.817	0.199	-3.206	3.927	.843
**Interactions**	MoP (July 2020) × Residence	0.071	3.266	0.022	-6.340	6.483	.983
	MoP (August 2020) × Residence	0.407	2.225	0.183	-3.960	4.774	.855
	MoP (September 2020) × Residence	1.570	2.195	0.715	-2.738	5.878	.475
	MoP (October 2020) × Residence	-0.267	1.822	-0.146	-3.843	3.310	.884
	MoP (November 2020) × Residence	-0.490	1.816	-0.270	-4.055	3.075	.787
	MoP (December 2020) × Residence	-0.338	1.818	-0.186	-3.907	3.232	.853
	MoP (January 2021) × Residence	-0.056	1.884	-0.029	-3.753	3.642	.976
	MoP (February 2021) × Residence	-0.345	1.819	-0.189	-3.915	3.226	.850
	MoP (March 2021) × Residence	-0.531	1.833	-0.290	-4.128	3.067	.772
	MoP (April 2021) × Residence (reference)	0[a]	0[a]	-	-	-	-
	MoP (July 2020) × COVID-19 IR	-0.339	-0.339	-0.578	-1.491	0.812	.563
	MoP (August 2020) × COVID-19 IR	-0.249	-0.249	-0.403	-1.460	0.962	.687
	MoP (September 2020) × COVID-19 IR	-0.359	-0.359	-0.606	-1.522	0.804	.545
	MoP (October 2020) × COVID-19 IR	-0.144	-0.144	-0.244	-1.307	1.018	.807
	MoP (November 2020) × COVID-19 IR	-0.343	-0.343	-0.583	-1.499	0.813	.560
	MoP (December 2020) × COVID-19 IR	-0.198	-0.198	-0.336	-1.353	0.957	.737
	MoP (January 2021) × COVID-19 IR	0.038	0.038	0.040	-1.797	1.872	.968
	MoP (February 2021) × COVID-19 IR	-0.070	-0.070	-0.110	-1.307	1.168	.912
	MoP (March 2021) × COVID-19 IR	-0.136	-0.136	-0.228	-1.307	1.035	.819
	MoP (April 2021) × COVID-19 IR (reference)	0[a]	0[a]	-	-	-	-

Abbreviations: MSBS-SF=Multidimensional State Boredom Scale-Short Form; RS-13=Resilience Scale (short form); MSPSS=Multidimensional Scale of Perceived Social Support; TILS=Three-Item Loneliness Scale; MoP=Month of Participation; IR=Incidence Rate; S.E.=standard error; LB=lower bound; UB=upper bound

[a] This parameter is set to zero because it is redundant.
