# Supplementary material for: Mental health in individuals with severe mental disorders during the covid-19 pandemic: a longitudinal investigation
Source: Schizophrenia (Heidelb). 2022 Mar 8;8(1):17. doi: 10.1038/s41537-022-00225-z (PMC8903129; doi:10.1038/s41537-022-00225-z)
Supplement: Supplementary file 2 — REPORTING SUMMARY [file 41537_2022_225_MOESM2_ESM.pdf]

nature portfolio

Corresponding author(s): Alex Hofer (NPJSCHZ-00753R)

Last updated by author(s): Nov 16, 2021

Reporting Summary

Nature Portfolio wishes to improve the reproducibility of the work that we publish. This form provides structure for consistency and transparency in reporting. For further information on Nature Portfolio policies, see our Editorial Policies and the Editorial Policy Checklist.

Statistics

For all statistical analyses, confirm that the following items are present in the figure legend, table legend, main text, or Methods section.

n/a	Confirmed	
☐	☒	The exact sample size (n) for each experimental group/condition, given as a discrete number and unit of measurement
☐	☒	A statement on whether measurements were taken from distinct samples or whether the same sample was measured repeatedly
☐	☒	The statistical test(s) used AND whether they are one- or two-sided<br>*Only common tests should be described solely by name; describe more complex techniques in the Methods section.*
☐	☒	A description of all covariates tested
☐	☒	A description of any assumptions or corrections, such as tests of normality and adjustment for multiple comparisons
☐	☒	A full description of the statistical parameters including central tendency (e.g. means) or other basic estimates (e.g. regression coefficient) AND variation (e.g. standard deviation) or associated estimates of uncertainty (e.g. confidence intervals)
☐	☒	For null hypothesis testing, the test statistic (e.g. F, t, r) with confidence intervals, effect sizes, degrees of freedom and P value noted<br>*Give P values as exact values whenever suitable.*
☒	☐	For Bayesian analysis, information on the choice of priors and Markov chain Monte Carlo settings
☒	☐	For hierarchical and complex designs, identification of the appropriate level for tests and full reporting of outcomes
☐	☒	Estimates of effect sizes (e.g. Cohen's d, Pearson's r), indicating how they were calculated

Our web collection on statistics for biologists contains articles on many of the points above.

Software and code

Policy information about availability of computer code

Data collection	Computer-based Health Evaluation System (CHES; version 5.27.1)<br>(Holzner et al. 2012; DOI: 10.1186/1472-6947-12-126)
Data analysis	IBM SPSS Version 27.0

For manuscripts utilizing custom algorithms or software that are central to the research but not yet described in published literature, software must be made available to editors and reviewers. We strongly encourage code deposition in a community repository (e.g. GitHub). See the Nature Portfolio guidelines for submitting code & software for further information.

Data

Policy information about availability of data

All manuscripts must include a data availability statement. This statement should provide the following information, where applicable:

- Accession codes, unique identifiers, or web links for publicly available datasets
- A description of any restrictions on data availability
- For clinical datasets or third party data, please ensure that the statement adheres to our policy

According to the Austrian and Italian law, data sharing requires approvals from the regional Committees for Medical and Health Research Ethics and from the regional Data Protection Officers. The data are therefore not publicly available. The data that support these findings can be provided by A.H., Medical University Innsbruck, upon reasonable request.

Field-specific reporting

Please select the one below that is the best fit for your research. If you are not sure, read the appropriate sections before making your selection.

☐ Life sciences ☒ Behavioural & social sciences ☐ Ecological, evolutionary & environmental sciences

For a reference copy of the document with all sections, see nature.com/documents/nr-reporting-summary-flat.pdf

Behavioural & social sciences study design

All studies must disclose on these points even when the disclosure is negative.

Study description	Quantitative longitudinal
Research sample	General population (342 f, 139 m; 44.3 +/- 13.6 years), SMI (27 f, 19 m; 48.9 +/- 14.3 years), MDD (41 f, 28 m; 48.5 +/- 13.9 years). Convenience sample from Tyrol (Austria) and South Tyrol (Italy).
Sampling strategy	Convenience sample. Sample size calculations were conducted with G*Power (version 3.1.9.2). They are based on the assumption of a type-one error probability of alpha = 0.05 and a power of 1 – beta = 0.8. Calculated f effect sizes have been transformed into η^2. Concerning repeated measures ANCOVA between factors analyses, with a total sample of 600 participants, effect sizes of $\eta^2 \geq 0.012$ can be detected. Effect sizes of $\eta^2 \geq 0.049$ (SMI vs. MDD), $\eta^2 \geq 0.011$ (SMI vs. controls), and $\eta^2 \geq 0.011$ (MDD vs. controls) are discoverable, when controls and patient groups are compared. For within factors analyses, effect sizes of $\eta^2 \geq 0.043$ (SMI group), $\eta^2 \geq 0.028$ (MDD group), and $\eta^2 \geq 0.004$ (control group) are detectable.
Data collection	Online survey using Computer-based Health Evaluation System (CHES; version 5.27.1) (Holzner et al. 2012; DOI: 10.1186/1472-6947-12-126)
Timing	In Tyrol, survey responses were collected between June 26th, 2020 and September 13th, 2020 (T1, baseline) and between November 30th, 2020 and January 24th, 2021 (T2, follow-up). In South Tyrol, survey responses were collected between September 7th, 2020 and November 22nd, 2020 (T1, baseline) and between February 8th, 2021 and April 4th, 2021(T2, follow-up).
Data exclusions	Control subjects reporting to have been diagnosed with a mental health disorder in the past as well as those who reported on current psychopharmacological and/or psychotherapeutic treatment were excluded from the analyses (N=449). Of the final sample (N=1,197), 481 individuals completed both baseline and follow-up surveys and are considered in this report.
Non-participation	1,542 patients diagnosed with SMI and 1,054 patients with MDD were invited by mail to participate, of which 190 enrolled. 46 out of 99 individuals in the SMI group and 69 out of 91 individuals in the MDD group completed both baseline and follow-up surveys and were included in the analyses of the current report. The control group was recruited through advertising in print media, email lists, flyer, and social media, and a total of 1,646 people participated in the baseline survey.
Randomization	---

Reporting for specific materials, systems and methods

We require information from authors about some types of materials, experimental systems and methods used in many studies. Here, indicate whether each material, system or method listed is relevant to your study. If you are not sure if a list item applies to your research, read the appropriate section before selecting a response.

Materials & experimental systems

n/a	Involved in the study
☒	☐ Antibodies
☒	☐ Eukaryotic cell lines
☒	☐ Palaeontology and archaeology
☒	☐ Animals and other organisms
☐	☒ Human research participants
☒	☐ Clinical data
☒	☐ Dual use research of concern

Methods

n/a	Involved in the study
☒	☐ ChIP-seq
☒	☐ Flow cytometry
☒	☐ MRI-based neuroimaging

Human research participants

Policy information about studies involving human research participants

Population characteristics	See above.
Recruitment	Local people who had been admitted to a psychiatric ward and had been diagnosed with a mental health disorder in 2019 as

Recruitment	well as a control group from the general population aged 18 and above were invited to complete an online survey. The control group was recruited through advertising in print media, email lists, flyer, and social media.
Ethics oversight	EC of the Medical University Innsbruck, EC of the Sanitary Agency of South Tyrol.

Note that full information on the approval of the study protocol must also be provided in the manuscript.
